# Frozen by stress: Stress increases scope insensitivity

**DOI:** 10.1371/journal.pone.0223489

**Published:** 2019-10-04

**Authors:** Lu Li, Yuanyuan Liu, Yuanyuan Jamie Li

**Affiliations:** 1 School of Governmant, University of Chinese Academy of Social Sciences, Beijing, P.R. China; 2 School of Management, Xi’an Jiaotong University, Xi’an, Shaanxi Province, P.R. China; 3 School of Business, Southern University of Science and Technology, Shenzhen, Guangdong Province, P.R. China; Mälardalen University, SWEDEN

## Abstract

Stress has become a widely experienced state all around the world, and previous literature has found that stress impacts individuals’ cognition, emotion, coping behaviors and psychological well-being in general. Relatively little is known about how stress influences individuals’ perception of stimuli changes, a ubiquitous phenomenon known as scope sensitivity. In the current work, we explore whether individuals with higher levels of chronic stress are sensitive to stimuli changes, such as price and quantity differences. Two empirical studies consistently show that chronically stressed individuals exhibit scope insensitivity, as they rated the expensiveness of two hotel rooms with different prices as being less different and indicated a smaller difference in their willingness-to-buy five CDs versus ten CDs. Possible explanations and theoretical and practical implications in the broader field are discussed.

## Introduction

“About eight in 10 Americans say they frequently (44%) or sometimes (35%) encounter stress in their daily lives. Just 17% say they rarely feel stressed, while 4% say they never do.”

-Gallup, 2017 (https://news.gallup.com/poll/224336/eight-americans-afflicted-stress.aspx)

According to the annual nationwide survey by the American Psychological Association in 2018, 91% of 3,458 Gen Z adults reported that stress has resulted in either physical or emotional symptoms, such as feeling depressed or sad (58%), or in a lack of interest, motivation or energy (55%). Importantly, the level of stress is increasing, and most people are living in a stressed state, with long work hours and commute times, a high uninsured percentage and unemployment rate, high living costs, etc. Due to the ubiquity of stress, stress should receive more attention in various research fields, including marketing, psychology, neuroscience, environment, and sociology [1].

According to Hsee and Rottenstreich (2004), scope variable represents “any quantitative aspect of a stimulus” [2], thus scope sensitivity could be regarded as individuals’ perceptual sensitivity to the quantitative changes of stimuli, including sensitivity to changes in price, quantity, risk, and resource valuation. Scope sensitivity has a large impact on our daily lives and society, especially on the valuation of both public goods and private goods [3, 4, 5]. With the rapid increase in stress levels, together with the rapid changes to society, it is important to understand how individuals react to changes in external stimuli. Therefore, in the current paper, we explore how stress influences scope sensitivity.

### Stress

Stress refers to a psychological state in which one experiences harm, a threat, or a challenge to their current resources and capacity [6]. Prior research has distinguished acute stress and chronic stress from the perspective of duration [1]. Acute stress is generated from discrete challenging events, while chronic stress often originates from continuously stressful situations in daily life or social environments [7, 8].

Although there is literature on the similar impacts that chronic stress and acute stress have on human well-being [8, 9], some researchers have found divergent impacts of chronic stress and acute stress on mammals and human beings [7, 10, 11]. From life event checklists analysis, Avison and Turner found that chronic stress is a significant contributor to individuals’ depressive symptoms, but the influence of acute stress on depression dissipates in a very short time [7]. This is consistent with Lazarus and DeLongis’s finding that chronic stress has a stronger impact on psychological adjustment than acute stress [11]. In contrast, the impact of acute stress on depression occurrence may depend on the existence of chronic stress, which is less influential on depression among individuals who are experiencing chronic stress [12]. Therefore, in this paper, we only explore how chronic stress influences scope sensitivity.

Chronic stress, a state associated with persistent distress, has been documented to increase negative emotion and impair human health and cognition [8, 13]. Exposure to chronic stressors, such as childhood poverty, was found to influence the amygdala and prefrontal cortex regions of the brain, which are the emotion regulatory systems, thus making people less likely to regulate their negative emotions [14, 15]. Several studies have shown that chronic stress increases addictive substance abuse, which impairs individuals’ well-being [9]. Employees with a high level of chronic work stress had a higher possibility of having metabolic syndrome [13].

In addition to impairing health and emotions, chronic stress could also cause dysfunctions in cognition, attention and memory [16, 17, 18, 19]. For instance, performance deficits were found for episodic memory, working memory, mental tempo, semantic access, as well as prospective memory among perceived chronic stress patients compared to controls [18]. Similarly, chronic stress was also found to cause worse performance in the Stroop task. Participants under chronic stress revealed more errors and longer reaction time [19]. In addition, it was found that chronic stress impaired attentional control and disrupted functional connectivity that mediates attention shift [17].

At the behavioral level, stressed individuals use both passive and active coping strategies. Rats and primates freeze and inhibit their behaviors when stressed [20, 21]. Regarding passive coping strategies, researchers found that in-store stress can terminate an on-going purchase among task-oriented consumers. However, for recreation-oriented consumers, in-store stress creates an inverted U-shape on purchase abandonment [22]. Regarding active coping strategies, stress enhances both financial savings and spending on necessities [23] because both savings and necessities can compensate for a lack of control. In this paper, we explore how stress influences scope sensitivity.

### Scope sensitivity

Scope sensitivity represents the level of individuals’ responsiveness towards changes in external cues, such as perceived price changes and perceived quantity changes [2, 24]. Several factors, have been documented to influence the perceived difference between a large and small scope for stimuli. Attention is needed to sense changes and differences in stimuli [25, 26]. When evaluating isolated individuals, individuals were found to be more scope insensitive (see a review) [27]. Moreover, people were more sensitive to the duration of a familiar stimulus but were insensitive to the duration of an unfamiliar stimulus [28].

Scope insensitivity is also influenced by goals and value orientations. For example, Pickett, Gardner, and Knowles showed that when the desire to belong was enhanced, individuals were more sensitive to human cues, such as facial expressions and vocal tones [29]. Moreover, Huang, Huang, and Jiang demonstrated that individuals became scope insensitive after exposure to death-related cues because death-related cues shifted individuals’ value focus from extrinsic to intrinsic [30].

Moreover, scope insensitivity may result from a reliance on affect [2, 24]. Hsee and Rottenstreich showed that when pandas were presented in an affect-rich way, participants indicated they would donate a similar amount of money to save one panda or four pandas, suggesting scope insensitivity [2]. However, when pandas were presented in simple dotes, participants became scope sensitive and donated significantly more money to four pandas than one panda. Building on and extending the above findings, Chang and Pham found that reliance on affect increased scope insensitivity in decisions that were psychologically proximate but not in decisions that were psychologically distant [24].

### Hypothesis

Two accounts could help us to form our prediction that chronic stress increases scope insensitivity. From the cognition perspective, previous literature has found that chronic stress impairs cognitive ability and also individual attention as shown in various cognitive tasks [16, 17, 18, 19]. From the affect perspective, chronic stress has been documented to increase negative emotions [14]. Negative emotions have been found to narrow attention scope [31] and lead to more self-focused attention [32] in existing literature. Thus, it is reasonable to infer that individuals in negative emotions allocate less attention to external stimuli and thus less sensitive to external stimuli, leading to scope insensitivity [25, 26, 30].

In summary, in the current paper, we speculate and expect to find that perceived chronic stress, accompanied by impaired cognitive ability and attention, as well as reliance on affect may increase scope insensitivity [2, 24]. That is:

H: Individuals with higher levels of perceived chronic stress are more scope insensitive.

## Study 1

### Materials and methods

#### Participants

One hundred ninety-nine participants (*M*_age_ = 34.84; SD = 10.82; 81 female) were recruited online from Amazon Mechanical Turk (mTurk) for a small participation reward.

This study was carried out in accordance with the recommendations of APA’s ethical guidelines, All subjects gave informed consent prior to participation in the study. All subjects could abort the experiment at any time.

#### Experimental design and procedure

First, all participants answered the 14-item scale [33] (α = 0.845), which is widely used in the literature and measures individuals’ perceived chronic stress. In the scale, participants indicated the frequency of 14 stress-related feelings or thoughts on a five-point scale (1 = “never”, 2 = “almost never”, 3 = “sometimes”, 4 = “fairly often”, and 5 = “very often”), with items such as “In the last month, how often have you felt difficulties were piling up so high that you could not overcome them?” and “In the last month, how often have you been angered because of things that happened that were outside of your control”. Frequency ratings were averaged to measure perceived stress (α = 0.89), and a higher score indicates a higher level of stress. Following the procedure from previous research on scope insensitivity [34], participants were instructed to rate the perceived expensiveness of a hotel room at the price of $138 (small scope) and $344 (large scope), anchored by “1-not expensive at all” and “9-every expensive”. The order of the hotel prices presented was counterbalanced. The difference of perceived expensiveness for a hotel at price of $138 or $344 represents the perceived difference between a small ($138) and large ($344) scope. Finally, demographic information, such as age, gender, education, income, and employment, was collected.

#### Results and discussion

Participants who rated $138 to be more expensive than $344 were excluded from further analysis, resulting in a final dataset of 174 participants.

**Fig 1:**
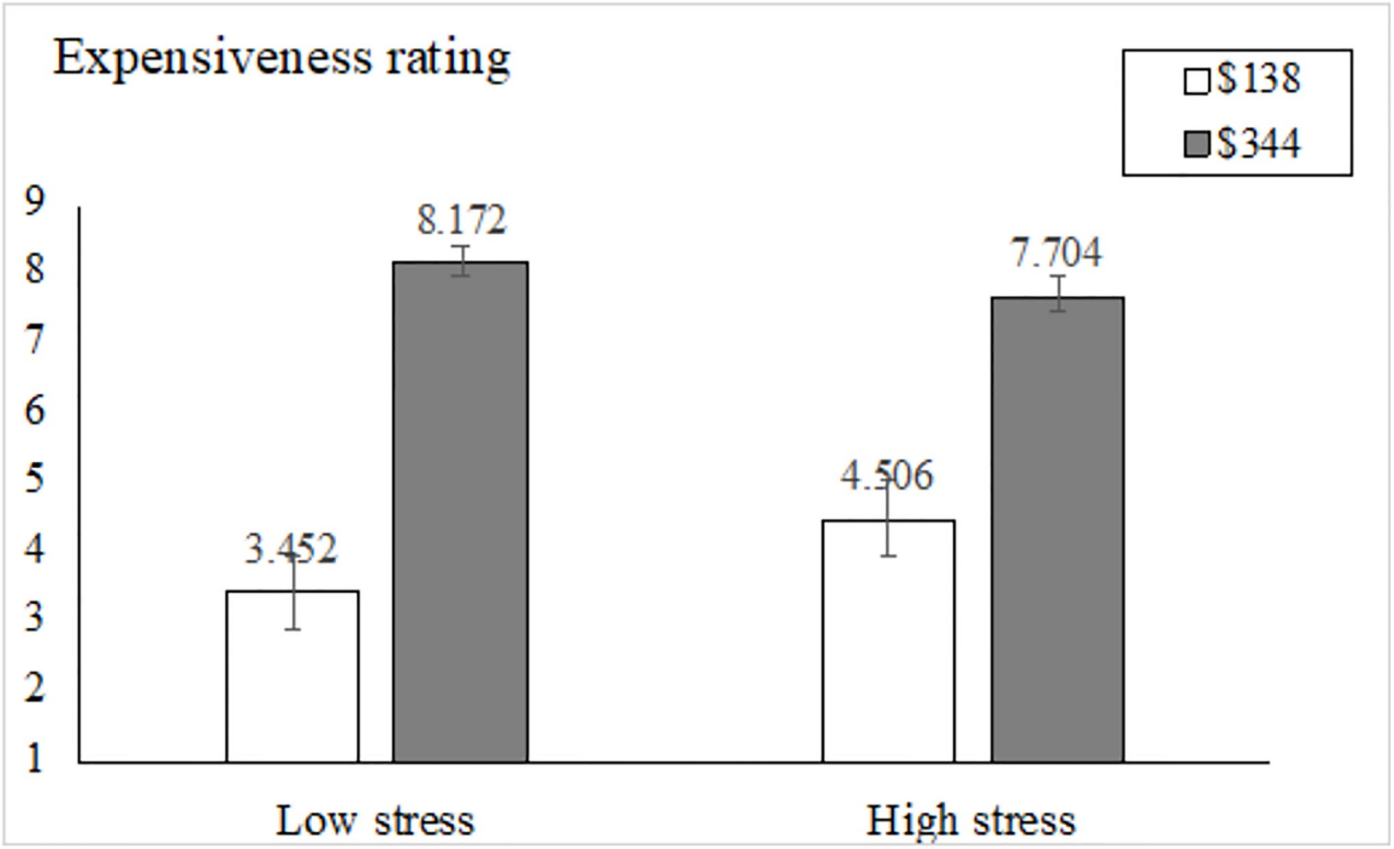
Stress and scope sensitivity in hotel room price evaluation.

Consistent with previous literature [30], repeated measures ANOVA was applied. Since the perceived chronic stress measure is a continuous variable, we performed a median split on stress scores and treated the expensiveness ratings for the prices $138 and $344 as repeated measures. First, the main effect of stress was not significant, *F*(1, 172) = 1.905, *p* = 0.169. Individuals with high levels of stress (*M*_*high_stress*_ = 6.105, SD = 1.937) did not rate the hotels to be more expensive than those with low stress (*M*_*low_stress*_ = 5.812, SD = 1.494) in general. However, the main effect of price was significant, *F*(1, 172) = 566.856, *p* < .001, η^2^ = 0.767; that is, participants rated hotel rooms with a price at $344 (*M*_*$344*_ = 7.954, SD = 1.29) to be significantly more expensive than those at $138 (*M*_*$138*_ = 3.943, SD = 2.22). Moreover, this main effect was qualified by stress level, *F*(1, 172) = 20.970, *p* < .001, η^2^ = 0.109 (Fig 1). Individuals under high levels of stress indicated the hotel room at $138 to be significantly more expensive, *F*(1, 172) = 10.309, *p* < .01, η^2^ = 0.057, but the hotel room at $344 to be significantly less expensive, than those under low levels of stress, *F*(1, 172) = 5.830, *p* = .017, η^2^ = 0.033. Consistent with our predictions, we conclude that scope sensitivity was smaller in individuals with high stress levels than in those with low stress levels.

Order effect was not found in this study, thus omitted in the formal analysis. Specifically, when order was added into the repeat-measure analysis as another independent variable, neither the main effect of order, *F*(1, 170) = 2.888, *p* = .091, η^2^ = 0.017, nor the three-way interaction of stress, order and repeated measure was significant, *F*(1, 170) = 0.004, *p* = .950.

Demographic variables, such as age and gender, did not influence our findings about the relationship between perceived chronic stress and scope. In the repeated measures analysis, when age was included as a covariate, the interaction between perceived chronic stress and price level was significant as well, *F*(1, 170) = 16.103, *p* < .001, η^2^ = 0.087; when including gender, the interaction between perceived chronic stress and price level stayed significant, *F*(1, 171) = 23.606, *p* < .001, η^2^ = 0.121; when both age and gender were included, the interaction between perceived chronic stress and price level was still significant, *F*(1, 169) = 18.225, *p* < .001, η^2^ = 0.097. Additionally, neither the main effect of age, *F*(1, 169) = 0.688, *p* = .408, nor gender, *F*(1, 169) = 0.121, *p* = .728, reached significance.

Alternatively, to further check the robustness of our findings, a linear regression was applied. To investigate within-subjects moderation/mediation effects, several empirical articles used the difference score between within-subjects measures as the dependent variable [35, 36]. Therefore, an expensiveness rating of $344 minus $138 was the new dependent variable, and a lower score indicated a higher level of scope insensitivity.

Model 1 of the Process [37] was applied to explore the impact of stress (standardized score) on scope sensitivity, with presentation order as a moderator (1 = ascending; 0 = descending). Consistent with our expectation, the main effect of stress was significant, coeff = -0.4968, se = 0.2197, t = -2.2616, *p* = .0250, 95% CI [-0.9304, -0.0632]. Participants with a higher level of stress were less sensitive to scope, and the perceived expensiveness difference between $138 and $344 was smaller. The main effect of order was marginally significant, coeff = -0.6625, se = 0.3377, t = -1.9618, *p* = .0514, 95% CI [—1.3292, 0.0041]. Participants were more sensitive to scope when prices were presented in descending order than ascending order. More importantly, the interaction between stress and order was not significant, t = -0.8746, *p* = .383. Thus, the order did not interfere with our findings.

## Study 2

Study 2 aims to replicate our findings in Study 1 with another dependent variable. To prevent a priming effect from participants answering the stress scale before the expensiveness rating, we measured scope sensitivity before the stress measure in Study 2.

### Materials and methods

#### Participants

Two hundred forty-eight participants (*M*_age_ = 32.00; SD = 9.52; 88 female) were recruited online from mTurk for a small participation reward.

This study was carried out in accordance with the recommendations of APA’s ethical guidelines, All subjects gave informed consent prior to participation in the study. All subjects could abort the experiment at any time.

#### Experimental design and procedure

The procedure was similar to Study 1, except for the order of the scales. In this study, a CD purchase scenario^14^ was adopted to measure scope sensitivity before the stress scale was presented. In the purchase scenario, participants were instructed to indicate their maximum willingness-to-pay (WTP) price for a bundle of five Beatles CDs and ten Beatles CDs, ranging from $0 to $100. The order that the 5 CDs and 10 CDs options were presented was counterbalanced. The same 14-item chronic stress scale (α = 0.602) as in Study 1 was applied.

#### Results and discussion

Unlike the expensiveness ratings in Study 1, the actual selling price for ten Beatles CDs is not always higher than that of five CDs in the marketplace. Therefore, repeated measures analysis [30] was adopted, with dichotomous stress levels as a between-subjects factor and the indicated WTP for five CDs and ten CDs as repeated measures. Neither the main effect of presentation order (*F*(1, 246) = 1.108, *p* = .294) nor the interaction with WTP was significant (*F*(1, 246) = 0.641, *p* = .424), thus they were omitted in the analysis.

**Fig 2:**
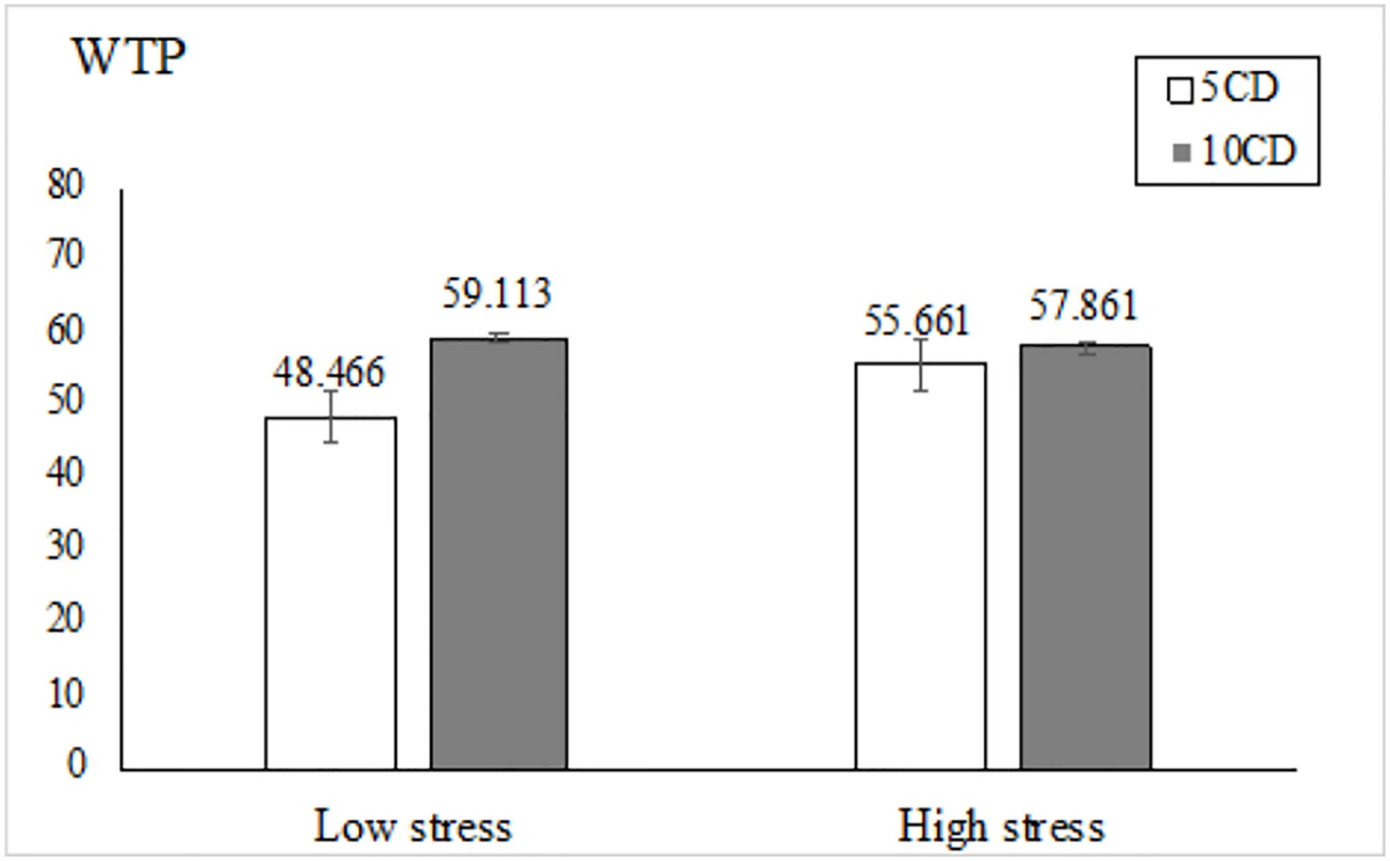
Stress and scope sensitivity in purchasing CDs.

Consistent with our findings in Study 1, repeated measures analysis revealed that the main effect of stress was not significant, *F*(1, 246) = 0.889, *p* = 0.347. However, the main effect of product quantity reached significance, *F*(1, 246) = 31.604, *p* < .001, η^2^ = 0.114. In general, participants would spend more on ten Beatles CDs (*M*_10CD_ = $58.532, SD = 26.077) than five Beatles CDs (*M*_5CD_ = $51.802, SD = 26.705). Moreover, the interaction between stress and product quantity was significant, *F*(1, 246) = 13.662, *p* < .001, η^2^ = 0.053 (Fig 2). Specifically, participants experiencing low levels of stress indicated a significantly higher WTP for ten CDs (*M*_10CD_ = $59.113, SD = 27.285) than five CDs (*M*_5CD_ = $48.466, SD = 25.899), *F*(1, 246) = 46.810, *p* < .001, η^2^ = 0.16. However, participants experiencing high stress levels were scope insensitive, indicating comparable WTP prices for both ten CDs (*M*_10CD_ = $57.861, SD = 24.708) and five CDs (*M*_5CD_ = $55.661, SD = 27.211), *F*(1, 246) = 1.728, *p* = 0.190. From another perspective, when indicating WTP for five CDs, highly stressed individuals indicated a significantly higher WTP than those experiencing less stress, *F*(1, 246) = 4.541, *p* = .034, η^2^ = 0.018. However, the WTP prices for ten CDs did not differ between the high and low stress groups, *F*(1, 246) = 0.142, *p* = 0.707.

Age and gender did not influence our findings regarding the relationship between perceived chronic stress and scope either. When age was included as a covariate, repeated measures analysis revealed that the interaction between perceived chronic stress and scope was significant, *F*(1, 245) = 14.194, *p* < .001, η^2^ = 0.055, as it was when gender was included as a covariate, *F*(1, 245) = 14.667, *p* < .001, η^2^ = 0.056. When both age and gender was included in the analysis, the interaction between perceived chronic stress and scope stayed the same, *F*(1, 244) = 15.516, *p* < .001, η^2^ = 0.060. Consistent with Study 1, neither the main effect of age, *F*(1, 244) = 0.012, *p* = .911, nor gender, *F*(1, 244) = 1.637, *p* = .202, was significant in the repeated-measure analysis.

## General discussion

In this paper, two empirical studies consistently showed that a perceived chronic stress state increased scope insensitivity. In Study 1, we found that individuals under high levels of stress indicated a hotel room at $344 to be significantly less expensive, but a hotel room at $138 to be significantly more expensive, than those experiencing low stress levels, suggesting scope insensitivity for price perception. In Study 2, the relationship between scope insensitivity and perceived chronic stress was replicated in a hypothetical purchase scenario with a different measurement order. Participants under stress indicated a significantly smaller WTP difference between a bundle of five Beatles CDs and a bundle of ten Beatles CDs.

Several other explanations related to acute stress may explain the results we found for chronic stress and scope insensitivity. First, our findings could be explained by reliance on system 1. The stress literature has shown that individuals under stress rely more on system 1 (see a review) [38], and reliance on affect could increase scope insensitivity [2, 24]. Second, attentional focus narrowing and mental resource depletion due to stress [39, 40] may lead to scope insensitivity, as well. Narrowed attentional focus and depleted mental resources may make stressed individuals allocate attention to the most important stimuli, thus becoming insensitive to less important stimuli, such as scope information. Future research is needed to directly show the underlying process for our findings.

### Limitations and implications

In this paper, two correlational studies were conducted to explore the relationship between perceived chronic stress and scope insensitivity. It is not confirmative from our findings that stress is the reason for scope insensitivity. Therefore, the causal relationship needs further exploration by directly manipulating stress. Second, all our data were collected online from Amazon Mechanical Turk, which only included participants from the United States. Future studies are needed to replicate the effect with participants in other cultures.

With the rapid increase in stress, increasingly more individuals are living under stress. Understanding the impact of stress on individuals’ decision making has practical implications for marketers, psychologists, designers and policymakers. Based on our findings, consumers may avoid making decisions when feeling stressed. However, it may be beneficial for marketers to increase prices or decrease product quantity among highly stressed consumers, as they are scope insensitive. Understanding that stressed individuals are less sensitive to scope assists psychologists in helping stress-related patients. Additionally, as stress levels increase, designers and policymakers may use more salient information about scope, as stressed individuals are less sensitive to scope.
