# Correction: Frozen by stress: Stress increases scope insensitivity

**DOI:** 10.1371/journal.pone.0224647

**Published:** 2019-10-28

**Authors:** The PLOS ONE Staff

There is an error in the Funding statement. The publisher apologizes for the error. The correct Funding statement is: This research was supported by the National Natural Science Foundation of China (URL: http://www.nsfc.gov.cn/) (71502080) to YJL, the National Natural Science Foundation of China (URL: http://www.nsfc.gov.cn/) (71702143) to YL, and Research Fund from the University of Chinese Academy of Social Sciences (URL: http://www.ucass.edu.cn/) (000720008) to LL. The funders had no role in study design, data collection and analysis, decision to publish, or preparation of the manuscript.
